# Insecticidal Activity of *Ricinus communis* Leaf Extracts Against *Bactrocera zonata* and *Bactrocera cucurbitae*: Identification of Potential Bioactive Compounds

**DOI:** 10.3390/insects17080811

**Published:** 2026-08-05

**Authors:** Rasheed Akbar, Sadia Manzoor, Irfana Lalarukh, Gul Makai, Somia Shehzadi, Asif Ali Khan, Ning Di, Jianfan Sun, Asmat Ullah, Jibiao Fan

**Affiliations:** 1 College of Animal Science and Technology, Yangzhou University, Yangzhou 225009, China; rasheed.akbar@uoh.edu.pk; 2 Department of Entomology, Faculty of Physical and Applied Sciences, The University of Haripur, Haripur 22062, Khyber Pakhtunkhwa, Pakistan; 3 Department of Botany, Government College Women University, Faisalabad 38000, Punjab, Pakistan; 4 Department of Zoology, Sardar Bahadar Khan Women University, Quetta 86400, Balochistan, Pakistan; 5 University Institute of Medical Laboratory Sciences, The University of Lahore, Lahore 54000, Punjab, Pakistan; 6 College of Food Science and Engineering, Shandong Agricultural University, Taian 271018, China; 7 Key Laboratory of Natural Enemies Insects, Ministry of Agriculture and Rural Affairs, Institute of Plant Protection, Beijing Academy of Agriculture and Forestry Sciences, Beijing 100097, China; 8 Institute of Environment and Ecology, School of Environment and Safety Engineering, Jiangsu University, Zhenjiang 212013, China; 9 Jiangsu Collaborative Innovation Center of Technology and Material of Water Treatment, Suzhou University of Science and Technology, Suzhou 215009, China; 10 College of Pharmaceutical Sciences, Zhejiang University of Technology, Hangzhou 310012, China

**Keywords:** *Bactrocera* spp., botanical insecticide, *Ricinus communis*, bioactive compounds, integrated pest management (IPM)

## Abstract

The melon fruit fly, *Bactrocera zonata*, and the pumpkin fruit fly, *Bactrocera cucurbitae* (Diptera: Tephritidae), are important agricultural pests that cause significant losses in a wide range of fruit and vegetable crops. Increasing concerns over pesticide resistance and environmental contamination have driven interest in plant-derived insecticide alternatives. This study investigated the insecticidal activity of *Ricinus communis* L. leaf extracts against both species. The crude leaf extract was fractionated using solvents of increasing polarity (n-hexane, methanol, and ethyl acetate). The resulting fractions were assessed for insecticidal activity, and the most active fraction was further purified using column chromatography. Chemical constituents were identified using gas chromatography–mass spectrometry (GC-MS) and Fourier transform infrared spectroscopy (FTIR). Bioassay results indicated that the methanol, n-hexane, and ethyl acetate fractions exhibited the highest insecticidal activity against both *B. zonata* and *B. cucurbitae*. GC-MS analysis revealed several bioactive constituents in the active fractions, including neophytadiene, fatty acid derivatives, and 11,14,17-eicosatrienoic acid, which was tentatively identified as a candidate constituent associated with the most active fractions. These findings suggest that *R. communis* leaf extracts contain bioactive constituents with insecticidal properties against tephritid fruit flies and may contribute to the development of plant-based pest management strategies. However, further work is required to isolate pure compounds and confirm their individual toxicological roles under field conditions.

## Simple Summary

This study evaluated the insecticidal potential of *R. communis* leaf extracts against the major agricultural pests *Bactrocera cucurbitae* (Coquillett), and the peach fruit fly *Bactrocera zonata* (Coquillett) (Diptera: Tephritidae). The methanol and ethyl acetate fractions demonstrated the highest bioactivity, and 11,14,17-eicosatrienoic acid was identified as a candidate constituent associated with mortality responses. Other compounds, such as neophytadiene and hentriacontane, showed comparatively lower activity. The findings suggest that *R. communis* extracts could contribute to plant-based pest management strategies. However, further research is needed to isolate pure compounds and validate their efficacy under field conditions.

## 1. Introduction

In Pakistan, more than 30 different types of vegetables are cultivated commercially, including cucurbits, solanaceous crops, and alliums [1]. However, the yield of these higher value vegetables is severely constrained by insect pests, particularly fruit flies belonging to the genus *Bactrocera* (Diptera: Tephritidae). The family Tephritidae comprises more than 4000 species distributed across temperate, subtropical, and tropical regions worldwide [2]. Among Tephritidae, the melon fruit fly *Bactrocera cucurbitae* (Coquillett), and the peach fruit fly *Bactrocera zonata* (Coquillett) are two of the most destructive pests of cucurbitaceous crops [3,4]. *B. cucurbitae* alone is responsible for yield reduction ranging from 30% to 100% depending on the cucurbit species, season, and infestation level [3]. Field surveys conducted in Pakistan have reported an infestation rate of 40% in bitter gourd (*Momordica charantia*) and 21% in bottle gourd (*Lagenaria siceraria*). In other cucurbit crops, the infestation rates range from 12.8% to 25.55% [5]. These fruit flies attacked more than 81 host plant species, including cucumber (*Cucumis sativus*), muskmelon (*Cucumis melo*), snake gourd (*Trichosanthes cucumerina*), and sponge gourd (*Luffa cylindrica*) [6].

In South Asia, the combined impact of both species *B. cucurbitae* and *B. zonata* on cucurbit production results in annual losses estimated at hundreds of millions of dollars [7]. Within integrated pest management (IPM) programs, despite their economic reports and severity, both species are often managed collectively. However, their relative susceptibility to botanical insecticides has rarely been systematically compared. Farmers have relied heavily on synthetic organophosphates, pyrethroids, and neonicotinoids to fruit flies’ management for decades [8,9]. The repeated and injudicious use of these chemicals has created several major problems although they are effective in the short term. First, it has resulted in the widespread development of insecticide resistance in tephritid populations [10]. Second, it has caused severe non-target toxicity to beneficial insects, pollinators, and soil microbiota [11]. Third, the accumulation of insecticide residues in food commodities and the environment has resulted in risks to human health [12]. For smallholder growers, the high costs of synthetic insecticides place a heavy financial burden on them. These growers constitute the majority of cucurbit producers in developing countries [13]. Therefore, there is an urgent and quick demand for eco-friendly, economically feasible, and resistance-breaking alternatives.

*Ricinus communis* L. (Euphorbiaceae), commonly known as the castor oil plant, is a fast-growing perennial shrub widely recognized for its diverse phytochemical profile and bioactive properties [14]. This plant species is native to Africa but is now established throughout tropical and subtropical regions of the world, including Brazil, India, South Africa, and Russia [15]. The seeds are well known for containing the highly toxic glycoprotein ricin, which has been extensively studied as a biological poison [16]. However, various bioactive compounds are found in the roots, stems and leaves. Phytochemical analyses of dried leaves of *R. communis* have revealed the presence of at least six flavones. Two alkaloids—namely, ricinine and N-demethylricinine—have also been identified, along with the several fatty acids and terpenes [17,18]. Seeds extracts of *R. communis* have exhibited larvicidal activity against mosquito vectors, including *Culex quinquefasciatus*, *Anopheles stephensi*, and *Aedes albopictus* [19]. Despite these promising reports, the insecticidal potential of *R. communis* leaf extracts against tephritid fruit flies, especially *B. cucurbitae* and *B. zonata,* has remained poorly explored.

Our previous study demonstrated that methanol, ethyl acetate, and n-hexane extracts of *R. communis* possess insecticidal activity against *B. zonata* and identified several bioactive constituents through FTIR, GC-MS, and multivariate analyses, with 11,14,17-eicosatrienoic acid emerging as a major candidate associated with toxicity. However, those findings were restricted to a single tephritid species, and therefore it remains unknown whether the observed insecticidal activity and solvent-dependent efficacy are conserved across other economically important fruit flies. Consequently, the broader applicability of *R. communis*-based botanical insecticides for tephritid pest management has not yet been established.

The melon fruit fly (*Bactrocera cucurbitae*) and peach fruit fly (*Bactrocera zonata*) differ considerably in their host preference, ecology, and physiological characteristics, which may influence their susceptibility to botanical insecticides [20]. Comparative toxicological evaluations of different solvent extracts against both species under identical experimental conditions have not previously been conducted. Such comparisons are important for identifying the most effective extraction solvent, determining species-specific differences in susceptibility, and assessing whether previously identified bioactive compounds provide consistent insecticidal activity across multiple economically important fruit fly species.

Therefore, the present study was designed to extend our previous findings by comparatively evaluating the insecticidal efficacy of methanol, ethyl acetate, and n-hexane extracts of *R. communis* leaves against both *B. cucurbitae* and *B. zonata*. Specifically, we aimed to (i) compare concentration- and time-dependent mortality responses of both fruit fly species, (ii) determine LC_50_ and LC_90_ values using probit analysis, (iii) characterize solvent-specific bioactive constituents using FTIR and GC-MS analyses, and (iv) identify the most effective extract and evaluate species-specific susceptibility to support the development of broad-spectrum botanical insecticides.

## 2. Materials and Methods

### 2.1. Rearing of Insect

Both fruit flies species, *B. cucurbitae* and *B. zonata,* were reared in cages (90 cm × 45 cm × 45 cm) on an artificial diet in the Laboratory of Entomology, The University of Haripur, during 2024. The species were identified by a specialist taxonomist in the department. The adults were fed in Petri dishes containing an artificial diet consisting of a mixture of protein hydrolysate (Protinex, Pfizer Ltd. Himachal Pradesh, India) and sugar (Dextrose-L) at a ratio of 1:1, and they were allowed to lay eggs on cucumber fruits placed inside the cage [10].

### 2.2. Collection of Plant Materials, Preparation of Ethanol Crude Extract, and Preparation of Test Solutions

Leaves of *R. communis* were collected from the University of Haripur. The leaves were washed with distilled water and air-dried at room temperature for 15 days. The dried leaves were then ground into fine powders according to [21,22]. Extractions were performed using a Soxhlet extractor in 80% ethanol for 8 h. The extracts were concentrated under reduced pressure at 60 °C, using a rotary evaporator in the Food Science laboratory. The resulting stock solution was stored at 4 °C until preparation [23,24]. For insecticidal bioassays, a 10% (*w*/*v*) stock solution of the crude ethanol extract was prepared and diluted with methanol, ethyl acetate, and *n*-hexane to obtain different solvent-based test solutions. For bioassays, the 10% stock solution was diluted separately with methanol, ethyl acetate, and *n*-hexane to prepare four concentrations (0.5%, 1.0%, 1.5%, and 2.0%) for each solvent preparation, resulting in a total of 12 treatment combinations (3 solvent preparations × 4 concentrations). Each treatment combination was tested with three independent replications, with 10 adults per replicate, for each species (Supplementary Table S1). These solutions were used to evaluate the contact toxicity of the crude ethanol extract against adults of *B. cucurbitae* and *B. zonata*. It is important to note that methanol, ethyl acetate, and n-hexane were used here solely as diluents to prepare the test concentrations from the crude ethanol extract, and not as primary extraction solvents. All bioassays for both species were conducted simultaneously in a single experiment using freshly prepared extracts and newly reared insect cohorts, following the protocols established in our previous work. It is important to note that the solvent-specific extracts used for chemical characterization (Section 2.4) were prepared independently from fresh plant material and were not derived from this ethanol extract.

### 2.3. Contact Toxicity Assay of Ethanol Crude Extract Solutions Against B. cucurbitae and B. zonata

The toxic potencies of ethanol crude extract solutions prepared in three solvents (methanol, ethyl acetate, and n-hexane) against both species were determined by exposing insects to four concentrations of 0.5%, 1.0%, 1.5% and 2.0%. Pure methanol, n-hexane or ethyl acetate was used as a control. The concentrations (0.5–2.0%) were selected based on preliminary range-finding experiments, where concentrations below 0.5% produced negligible mortality (<5%), and concentrations above 2.0% resulted in reduced solubility and excessive solvent effects. This concentration range is also consistent with previous studies on botanical extracts against tephritid fruit flies [1,10,25], allowing for meaningful comparison with the existing literature. Contact toxicity assays were conducted to determine insecticidal activity against adults of both species. Individuals of each species were chilled for 1 min at −2 °C. Using a camel-hair brush, each immobilized insect was transferred to a 9 cm Petri dish. Subsequently, 1.00 μL of each concentration of ethanol crude extract solution prepared in methanol, ethyl acetate, or n-hexane was applied to the insects’ dorsal thorax using a microliter syringe (Hamilton 700, Bonaduz, Switzerland). The adult stage was selected for evaluation because adult fruit flies are the primary dispersive and reproductive stage responsible for crop damage through oviposition. Contact toxicity is the most direct and widely used initial screening method for evaluating botanical insecticides [25].

Each experimental unit consisted of one Petri dish containing 10 adults, and three independent Petri dishes were used as biological replicates. As shown in Equation (1), the number of dead insects was counted after 24, 48 and 72 h of treatment. A completely randomized design (CRD) with three replications was used for the experiments. For each species, 450 insects were tested in total (360 treated + 90 controls). For each solvent-based treatment, the corresponding pure solvent (methanol, ethyl acetate, or *n*-hexane) was used as the control. Mortality in the corresponding solvent control ranged from 0% to 15% during the experimental period and was corrected using the Schneider–Orelli (1947) formula before statistical analysis [26],

(1) $$\mathrm{Corrected}\;\mathrm{Mortality}=\frac{\mathrm{Mortality}\left(\%\right)\mathrm{in}\;\mathrm{treatment}-\mathrm{Mortality}(\%)\;\mathrm{in}\;\mathrm{control}}{100-\mathrm{Mortality}(\%)\;\mathrm{in}\;\mathrm{control}}\times 100$$

### 2.4. Preparation of Solvent-Specific Extracts for Chromatographic Analysis

These solvent-specific extracts were prepared separately from the diluted ethanol extract used in the bioassays (Section 2.2) and were used for chromatographic characterization and subsequent fraction preparation. For chromatographic separation and chemical characterization, separate solvent-specific extracts of *R. communis* leaves were prepared using the solid–liquid extraction method [27]. The powdered plant material (20 g) was extracted separately with three different solvents: methanol, ethyl acetate, and *n*-hexane (100 mL each) in 500 mL conical flasks. The mixtures were agitated on an orbital shaker at 150 rpm for 2 h and subsequently kept at room temperature for 72 h to ensure complete extraction. Each extract was filtered, and the collected filtrates were concentrated using a rotary evaporator under reduced pressure. The dried solvent-specific extracts were stored at 4 °C until further chromatographic separation and fraction preparation. These extracts are hereafter referred to as the methanol extract, ethyl acetate extract, and *n*-hexane extract of *R. communis*.

### 2.5. Column Chromatography and Fraction Collection

Column chromatography was performed using a glass column (600 × 15 mm) packed with silica gel (60–120 mesh, Merck Darmstadt, Germany) as the stationary phase [28]. The crude methanol, ethyl acetate, and n-hexane extracts of *R. communis* were separately loaded onto the column and eluted using solvent systems of increasing polarity. Three major fractions (F1–F3) were obtained from each solvent-specific extract. Each collected fraction was concentrated using a rotary evaporator and subjected to thin-layer chromatography (TLC) analysis to evaluate separation patterns and purity.

TLC was performed on precoated silica gel 60 F_254_ aluminum plates using chloroform:n-hexane (50:50, *v*/*v*) and ethyl acetate:methanol:chloroform solvent systems as mobile phases. The developed plates were air-dried and visualized under UV light (254 nm). The retention factor (Rf) values were calculated for each separated component using Equation (2). The TLC profiles and Rf values of methanol, ethyl acetate, and n-hexane fractions are provided in Supplementary Tables S2–S4, respectively. The purified fractions were stored in sealed amber vials at 4 °C until further insecticidal activity evaluation.

(2) $$\mathrm{Rf}=\frac{\mathrm{Distance}\;\mathrm{travelled}\;\mathrm{by}\;\mathrm{compound}}{\mathrm{Distance}\;\mathrm{travelled}\;\mathrm{by}\;\mathrm{solvent}\;\mathrm{front}}$$

Fractions showing similar TLC profiles were pooled and considered for subsequent insecticidal activity evaluation [24].

### 2.6. Insecticidal Activity of Chromatographic Fractions Against B. cucurbitae and B. zonata

This bioassay was conducted as an independent experiment from the crude extract bioassays using freshly emerged flies. Contact toxicity bioassays were conducted to evaluate the insecticidal activity of chromatographic fractions obtained from the solvent-specific extracts of *R. communis* against adults of *B. cucurbitae* and *B. zonata*. Three chromatographic fractions (F1, F2, and F3) were obtained separately from each solvent-specific extract (methanol, ethyl acetate, and *n*-hexane) and evaluated individually. Each dried fraction was dissolved in its corresponding solvent and diluted to obtain four concentrations (0.5%, 1.0%, 1.5%, and 2.0%, *w*/*v*). The corresponding pure solvents were used as solvent controls. Each fraction concentration was tested using three independent replications, with 10 adults per replicate [10]. Adult flies were immobilized by chilling at −2 °C for 1 min and individually transferred to 9 cm Petri dishes using a camel-hair brush. A 1 μL dose of each fraction solution was applied to the dorsal thorax of individual insects using a Hamilton microsyringe (Hamilton 700, Bonaduz, Switzerland). Mortality was recorded after 24, 48, and 72 h of exposure.

### 2.7. Fourier-Transform Infrared Spectroscopy (FTIR)

FTIR spectroscopy was performed to identify functional groups present in the solvent-specific extracts of *R. communis* prepared using methanol, ethyl acetate, and n-hexane extraction (Section 2.4). The *R. communis* extracts were lyophilized after being frozen at −80 °C. The lyophilized extract’s infrared absorption spectra were observed in the 4000–400 cm^−1^ range using an FTIR spectrophotometer (Billerica, MA, USA) [29].

### 2.8. Gas Chromatography–Mass Spectrometry Analysis (GC-MS)

The solvent-specific extracts of *R. communis* prepared by direct extraction of leaf powder with methanol, ethyl acetate, and n-hexane (Section 2.4) were subjected to GC–MS analysis to identify their chemical constituents. The ethanol crude extract solutions used in the bioassays and the chromatographic fractions obtained in Section 2.5 were not analyzed by GC–MS. The extracts were analyzed using Shimadzu’s GC-MS QP2010 series (Kyoto, Japan) in electron impact ionization mode and a Bpx5 GC column (length, 30 m; film thickness, 0.25 µm; diameter, 0.25 mm). Helium gas (99.999%) was utilized as the carrier gas at a constant flow rate of 1 mL/min, and samples were injected in the split mode with a split ratio of 10:1. The temperature of the injector was 250 °C, and that of the ion source was 200 °C. The mass spectrophotometer was set up in positive electron ionization mode with an ionization energy of 70 eV, and the solvent delay was changed from 0 to 45 min. The fragmented scan interval ranged from *m*/*z* 35 to 500 Dm. Each component’s relative percentage was calculated using the peak area/total peak area ratio. The software used was Lab Solution (Shimadzu, Kyoto, Japan), and the library was the NIST Coin 4.0 library (National Institute of Standards Technology). All identifications were based on NIST library matching without analytical standards and should be considered tentative [30].

### 2.9. Statistical Analyses

Corrected mortality values were calculated using the Schneider–Orelli (1947) formula (Equation (1)). For each treatment concentration and exposure period, mortality data from three independent replicates, with 10 insects per replicate, were used for statistical analysis. A univariate ANOVA was performed to evaluate the effects of treatment on corrected mortality, followed by Fisher’s protected least significant difference (LSD) test for pairwise comparisons at a significance level of α = 0.05 [31]. Mortality data at each observation time (24, 48, and 72 h) were analyzed separately because LC_50_ and LC_90_ values were estimated independently for each exposure period. LC_50_ and LC_90_ values for *B. zonata* and *B. cucurbitae* were estimated using log-probit analysis in SPSS version 20, and the corresponding probit slopes and their standard errors (SE) were calculated from the fitted dose–response models. The resulting LC_50_ and LC_90_ estimates were interpreted in relation to the tested concentration range and observed mortality responses [32]. The goodness of fit of each probit model was assessed using Pearson’s chi-square (χ^2^) goodness-of-fit test. The null hypothesis (H_0_) was that the observed mortality responses did not differ significantly from those expected under the fitted probit model. A *p*-value > 0.05 indicated that H_0_ could not be rejected, suggesting no statistically significant lack of fit between the observed and expected mortality responses, whereas a *p*-value < 0.05 indicated a statistically significant lack of fit. Multivariate analysis was conducted using hierarchical cluster analysis (HCA) to compare the chemical profiles of the three solvent extracts based on the relative abundance profiles of the identified compounds. HCA was performed using complete linkage and Euclidean distance, and the resulting clustering patterns were visualized as a heat map using the Bioinformatics online platform. The concentrations of frequently occurring compounds in each solvent extract were calculated by multiplying the relative abundance of each compound by the corresponding extract dose. All statistical analyses, including ANOVA, probit analysis, and estimation of LC_50_, LC_90_, and probit slopes, were performed using SPSS version 20, whereas HCA and heat-map visualization were conducted using the Bioinformatics online platform www.bioinformatics.com.cn.

## 3. Results

The insecticidal activity of methanol, n-hexane, and ethyl acetate extracts of *R. communis* was evaluated against *B. zonata* and *B. cucurbitae*. The evaluation was conducted at four different concentrations (0.5%, 1.0%, 1.5%, and 2.0%) over three exposure periods: 24, 48, and 72 h. Mortality data and probit analysis parameters are summarized in Table 1, Table 2 and Table 3, respectively.

### 3.1. Methanol Extract

#### 3.1.1. Concentration- and Time-Dependent Mortality

The methanol extracts of *R. communis* exerted a clear concentration-dependent increase in mortality for both tested fly species at all exposure times (Table 1). The mortality of *B. zonata* was enhanced from 6.66% at 0.5% concentration to 23.33% at 2.0% concentration after 24 h. Meanwhile, *B. cucurbitae* exhibited higher observed mortality at the tested concentrations ranging from 20.00% to 46.00%. The LSD values for both species confirmed that these concentration effects were statistically significant (*p* < 0.05). Control mortality remained zero throughout the first 48 h. Mortality increased with increasing exposure time. *B. zonata* mortality reached 30.00% at the maximum concentration, whereas *B. cucurbitae* mortality reached 56.67% at 48 h of exposure. At the 2% concentration, higher mortalities of 43.33% (*B. zonata*) and 61.54% (*B. cucurbitae*) were observed after 72 h exposure. Control mortality remained negligible throughout the experiment and did not differ significantly from zero (*p* > 0.05).

#### 3.1.2. Lethal Concentration Dynamics

The estimated LC_50_ values of the methanol extract generally decreased with an increasing exposure time, suggesting increased toxicity following prolonged exposure. For *B. zonata*, the estimated LC_50_ decreased from 1.12% at 24 h to 0.50% at 48 h and 0.30% at 72 h. For *B. cucurbitae*, the corresponding estimates were 5.72%, 2.94%, and 1.38%, respectively. However, the 24 and 48 h LC_50_ estimates for *B. cucurbitae* were above the tested concentration range of 0.5–2.0% and therefore represent extrapolated estimates. The 72 h estimate was within the tested range. Accordingly, the apparent lower susceptibility of *B. cucurbitae* based on the 24 and 48 h LC_50_ estimates should be interpreted cautiously.

The estimated LC_90_ values were substantially more uncertain than the LC_50_ estimates. Several LC_90_ values were considerably above the highest tested concentration of 2.0%, including 35.56% for *B. zonata* at 24 h and 14.96–22.80% for *B. cucurbitae* across exposure times. These values therefore represent substantial extrapolations beyond the experimental concentration range and should not be interpreted as experimentally demonstrated concentrations required to achieve 90% mortality. Because 90% mortality was not consistently bracketed by the tested concentrations, LC_90_ estimates were considered unreliable for biological interpretation.

#### 3.1.3. Slope and Model Reliability

Slope values differed between species at each exposure time. For *B. zonata*, slopes were consistently steep: 4.6 ± 0.44 (24 h), 3.40 ± 0.38 (48 h), and 3.74 ± 0.35 (72 h). This shows a rapid increase in mortality with small increases in concentration. The slopes were approximately 1.20 ± 0.44 (24 h), 1.05 ± 0.38 (48 h), and 1.05 ± 0.35 (72 h) for *B. cucurbitae*. These near-unity slopes reflect a relatively shallow concentration response relationship. Mortality increases gradually over the tested concentration range in this relationship. The shallow slopes observed for *B. cucurbitae* indicate a more gradual increase in mortality across the tested concentration range. However, the steep slopes in *B. zonata* reveal higher sensitivity to concentration changes compared to *B. curcubitae*.

#### 3.1.4. Species Comparison

Overall, mortality increased with both concentration and exposure time in the two fruit fly species. Although *B. cucurbitae* exhibited higher observed mortality than *B. zonata* at several equivalent concentrations, the estimated LC_50_ values were consistently lower for *B. zonata*. This difference indicates that mortality at individual concentrations and probit-derived lethal concentration estimates describe different aspects of toxicity. Therefore, species susceptibility was interpreted based on both observed mortality and LC estimates.

### 3.2. N-Hexane Extract

#### 3.2.1. Concentration- and Time-Dependent Mortality

The n-hexane extract was significantly more potent than the methanol extract for both tested fly species (Table 2). The mortality of *B. zonata* ranged from 20.00% (0.5% concentration) to 48.00% (2.0% concentration), whereas *B. cucurbitae* mortality ranged from 13.33% to 53.33% after 24 h exposure. These values were approximately twice those observed with methanol at equivalent concentrations. LSD values also confirmed significant concentration-dependent effects. The difference become even more evident at 48 h of exposure. The mortality of *B. cucurbitae* reached 93.00% at the 2.0% concentrations, approaching complete mortality. After 72 h of exposure, *B. cucurbitae* achieved 100.00% mortality at 2.0% concentration, while *B. zonata* reached 86.60%. The mortality difference between both species at 72 h was non-significant, confirming that the observed effects were treatment-related.

#### 3.2.2. Lethal Concentration Dynamics

The estimated LC_50_ values of the n-hexane extract decreased with increasing exposure time in both species. For *B. zonata*, LC_50_ decreased from 2.18% at 24 h to 1.22% at 48 h and 0.65% at 72 h. For *B. cucurbitae*, LC_50_ decreased from 2.04% to 0.67% and 0.33% at 24, 48, and 72 h, respectively. The 24 h LC_50_ estimates for both species were slightly above the highest tested concentration of 2.0% and therefore represent extrapolated estimates, whereas the later estimates fell within or below the tested range. At 72 h, the lower LC_50_ estimate for *B. cucurbitae* than for *B. zonata* was consistent with the higher observed mortality of *B. cucurbitae* at the upper concentrations.

LC_90_ estimates were more uncertain, particularly where they exceeded the experimental concentration range. For example, the estimated LC_90_ for *B. zonata* was 20.70% at 24 h and 15.60% at 48 h, whereas the corresponding values for *B. cucurbitae* were 8.90% and 1.81%, respectively. Because several of these estimates were extrapolated beyond the maximum tested concentration of 2.0%, they were not used as the primary basis for comparing insecticidal potency. At 72 h, the observed mortality at 2.0% reached 90.00% for *B. zonata* and 100.00% for *B. cucurbitae*, providing stronger experimental evidence of high activity than the extrapolated LC_90_ estimates.

#### 3.2.3. Slope and Model Significance

Slope values differed between species at each exposure time. At 24 h, the slopes values were 1.20 ± 0.45 for *B. zonata* and 2.00 ± 0.45 for *B. cucurbitae*. At 48 h, the slopes values were 1.04 ± 0.34 and 3.00 ± 0.34, for *B. zonata* and for *B cucurbitae* respectively. At 72 h, the slopes values were 1.50 ± 0.31 for *B. zonata* and 2.60 ± 0.31 for *B. cucurbitae*. For *B. cucurbitae*, slopes remained consistently above 2.0, indicating a relatively steep concentration–response relationship at all time points. For *B. zonata*, slopes remained within the range of 1.0–1.5, reflecting a shallower response in which mortality increased more gradually with increasing concentration. For *B. cucurbitae*, the probit model was highly significant at all time points (*p* = 0.00, 0.00, and 0.02), confirming strong dose–response relationships. For *B. zonata,* the model was significant at 72 h (*p* = 0.0432), indicating that prolonged exposure improved the predictability of concentration effects.

#### 3.2.4. Species Susceptibility Comparison

The *n*-hexane extract exhibited strong insecticidal activity against both fruit fly species. Although mortality increased progressively with concentration and exposure time in both species, *B. cucurbitae* was more susceptible after prolonged exposure, as indicated by its lower LC_50_ and LC_90_ values at 48 and 72 h compared with *B. zonata*.

### 3.3. Ethyl Acetate Extract

#### 3.3.1. Concentration- and Time-Dependent Mortality

The ethyl acetate extract showed intermediate insecticidal potency between the methanol and n-hexane (Table 3). At 24 h, mortality of *B. zonata* ranged from 20.00% to 46.00%, whereas, mortality of *B. cucurbitae* ranged from 13.33% to 46.66%. The LSD values (18.828 for *B. zonata,* and 25.45 for *B. cucurbitae*) confirmed a significant concentration-dependent effect (*p* < 0.05). At 48 h, mortality reached 58.00% for *B. zonata* and 59.86% for *B. cucurbitae* at the 2.0% maximum concentration. At 72 h, the maximum mortality was 67.00% for *B. zonata,* and 86.53% for *B. cucurbitae* at 2.0% concentration. The control mortality for *B. zonata* at 72 h was 13.00%, which was not statistically significant.

#### 3.3.2. Lethal Concentration Dynamics

For *B. zonata*, the estimated LC_50_ decreased from 3.02% at 24 h to 1.83% at 48 h and 1.28% at 72 h. For *B. cucurbitae*, LC_50_ decreased from 2.55% to 1.61% and 0.92% at 24, 48, and 72 h, respectively. The 24 h LC_50_ estimates for both species exceeded the highest tested concentration of 2.0% and therefore represent extrapolations, whereas the later estimates were within the tested range. The progressive decline in LC_50_ estimates with exposure time was consistent with increasing mortality observed at longer exposure periods.

The LC_90_ estimates were substantially above the tested concentration range in several cases, particularly for *B. zonata*. For example, the estimated LC_90_ values were 40.10%, 24.30%, and 7.32% at 24, 48, and 72 h, respectively. For *B. cucurbitae*, the corresponding estimates were 15.70%, 5.87%, and 3.18%. Because these estimates were obtained largely through extrapolation beyond the maximum tested concentration of 2.0%, they were considered uncertain and were not used as the principal basis for interpreting species susceptibility. Instead, comparative interpretation was based primarily on observed mortality within the tested concentration range and LC_50_ estimates that were not substantially extrapolated.

#### 3.3.3. Slope and Model Significance

The slope values varied differed between the two species. For *B. zonata*, slopes were 1.14 ± 0.45 at 24 h, 1.04 ± 0.34 at 48 h, and 1.42 ± 0.31 at 72 h. For *B. cucurbitae*, slopes were 1.52 ± 0.45 at 24 h, 1.97 ± 0.34 at 48 h, and 1.71 ± 0.31 at 72 h. The consistently steeper slopes for *B. cucurbitae* indicate a more homogenous and abrupt increase in mortality with increasing concentration than in *B. zonata*. For *B. zonata,* the probit model was significant only at 72 h (*p* = 0.041). In contrast, the probit model for *B. cucurbitae* was highly significant at all time points. The lower significance for *B. zonata* reflects greater variability in response, particularly at shorter exposure times.

#### 3.3.4. Species Susceptibility Comparison

The ethyl acetate extract showed intermediate toxicity compared with the methanol and *n*-hexane extracts. Based on LC_50_ and LC_90_ values, *B. cucurbitae* exhibited greater susceptibility than *B. zonata*, particularly after 72 h of exposure. The lower LC values of *B. cucurbitae* indicate that this species required lower concentrations of ethyl acetate extract to achieve comparable mortality levels; this reduced susceptibility may be associated with species-specific physical characteristics such as more efficient detoxification mechanisms or differences in cuticular permeability, although these mechanisms were not directly investigated in the present study. The consistently probit slopes for *B. cucurbitae* across all time indicate a more uniform concentration mortality response than that of *B. zonata*, suggesting that ethyl-acetate-based formulations may provide a reliable control of this species.

### 3.4. R. communis Different Fraction Mortalities in Different Solvents Against B. cucurbitae and B. zonata

**Figure 1:**
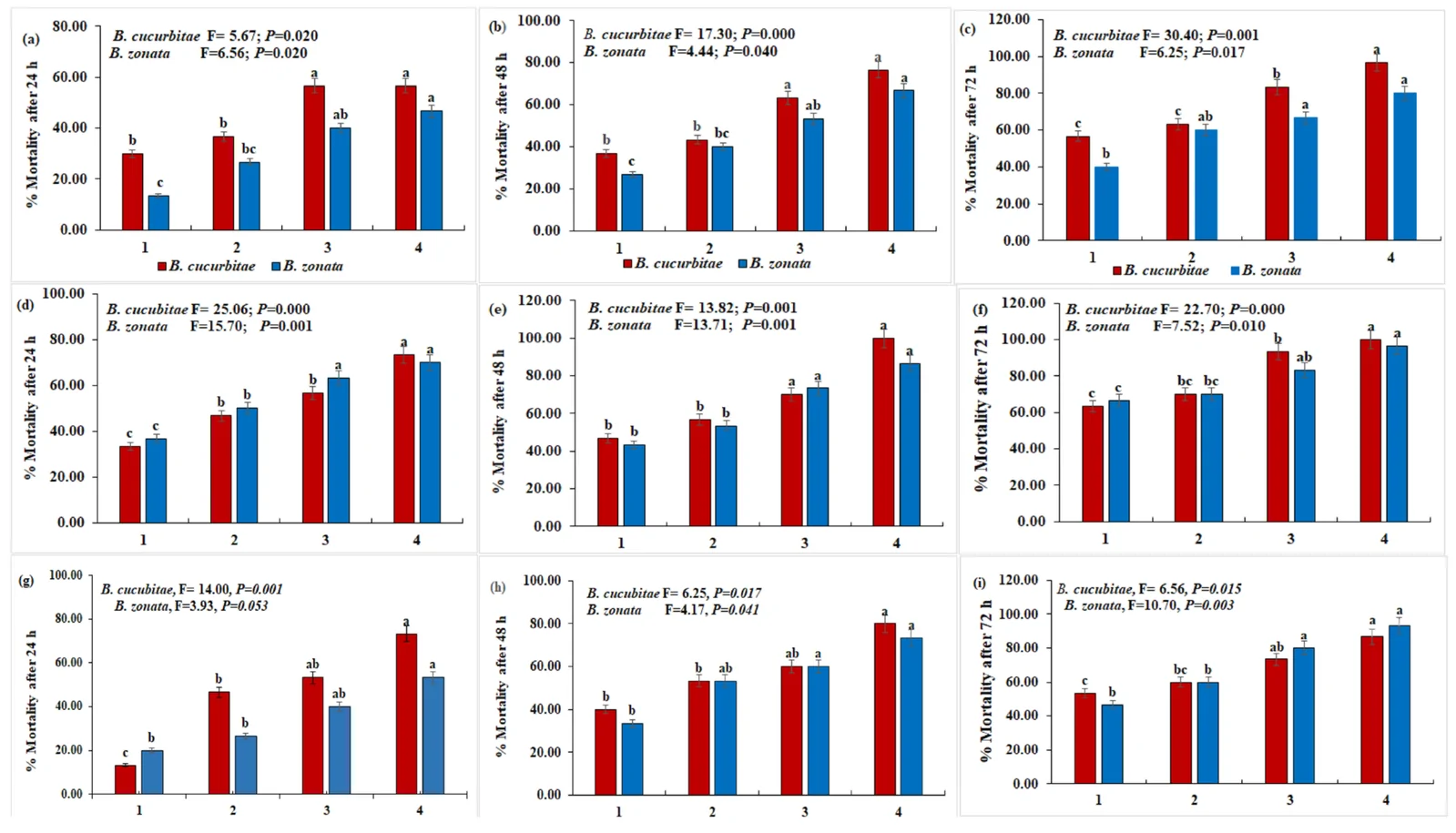
Mortality of *B. cucurbitae* (red bars) and *B. zonata* (blue bars) exposed to four concentrations (0.5%, 1.0%, 1.5%, and 2.0%, *w*/*v*) of *R. communis* chromatographic fractions. Panels (**a**–**c**) represent fraction F1 (methanol), panels (**d**–**f**) represent fraction F2 (n-hexane), and panels (**g**–**i**) represent fraction F3 (ethyl acetate). For each fraction, panels show mortality at 24 h (**a**,**d**,**g**), 48 h (**b**,**e**,**h**), and 72 h (**c**,**f**,**i**). On the X-axis, numbers 1, 2, 3, and 4 indicate 0.5%, 1.0%, 1.5%, and 2.0% concentrations, respectively. Different lowercase letters above bars denote significant differences among concentrations within each insect species and time point (Tukey’s LSD test, *p* < 0.05).

The insecticidal activity of *R. communis* extracts obtained using methanol, n-hexane, and ethyl acetate against *B. cucurbitae* and *B. zonata* was evaluated at four concentrations (0.5, 1, 1.5, and 2%) over periods of 24, 48, and 72 h as showed in Figure 1. Across all solvent extracts, insect species and exposure time mortality increased consistently with concentration and exposure duration.

For methanol fractions (panel a–c), after 24 h (panel a), mortality ranged from 30.00% at 0.5% to 56.67% at 2% in *B. cucurbitae* and from 13.33% to 46.67% in *B. zonata.* Concentration had a significant effect on mortality in both species (*B. cucurbitae*: F = 5.67, *P* = 0.022; *B. zonata*: F = 5.67, *P* = 0.023). At 48 h (panel b), mortality increased to 36.67–76.67% in *B. cucurbitae* and 26.67–66.67% in *B. zonata* with significant concentration effects for both species (*B. cucurbitae*: F = 17.3, *P* = 0.0007 and *B. zonata*: F = 17.3, *P* = 0.001 respectively). By 72 h (panel c), the highest concentration (2.0%) resulted in 96.67% mortality in *B. cucurbitae* and 80.00% in *B. zonata.* Mortality remained significantly affected by concentration (*B. cucurbitae*: F = 30.4, *P* = 0.001; *B. zonata*: F = 16.25, *P* = 0.0172). Across all time points, *B. cucurbitae* was consistently showed more susceptible to the methanol extract than *B. zonata*.

For N-hexane fractions (panels d–f), among the test solvents, n-hexane exhibited the strongest insecticidal activity. At 24 h (panel d), mortality ranged from 33.33 to 73.33% in *B. cucurbitae* and from 36.67 to 70.00% in *B. zonata* with significant effects in both species (*B. cucurbitae*: F = 25.06, *P* = 0.000; *B. zonata*: F = 15.7, *P* = 0.001). After 48 h (panel e), the 2% higher concentration resulted in 100.00% mortality in *B. cucurbitae* and 86.67% in *B. zonata* with significant concentration-dependent differences in both species (*B. cucurbitae*: F = 13.8, *P* = 0.001; *B. zonata*: F = 13.7, *P* = 0.001). At 72 h (panel f), complete mortality (100.00%) was recorded for *B. cucurbitae*, and 96.67% for *B. zonata* at 2% concentration with significant concentration effects (*B. cucurbitae*: F = 22.7, *P* = 0.000; *B. zonata*: F = 7.52, *P* = 0.010). Even the lowest concentration (0.5%) gave >63% mortality after 72 h exposure.

For ethyl acetate extracts (panels g–i), moderate but significant insecticidal activity was observed. At 24 h (panel g), mortality ranged from 13.33% to 73.33% in *B. cucurbitae* and 20.00% to 53.33% in *B. zonata,* with significant concentration-dependent differences in both species (*B. cucurbitae*: F = 14.0, *P* = 0.001; *B. zonata*: F = 6.25, *P* = 0.017). At 48 h (panel h), the 2% concentration resulted in 80.00% and 73.33% mortality, respectively, with significant concentration-dependent differences in both species (*B. cucurbitae*: F = 10.7, *P* = 0.003; *B. zonata*: F = 6.56, *P* = 0.015). By 72 h (panel i), mortality reached 86.67% in *B. cucurbitae* and 93.33% in *B. zonata* at 2% concentration, with significant concentration effects (*B. cucurbitae*: F = 6.56, *P* = 0.015; *B. zonata*: F = 10.7, *P* = 0.003). The ethyl acetate extracts showed more efficacy against *B. zonata* at the highest concentration and longest exposure period.

In simple words, all three solvent-derived fractions of *R. communis* exhibited significant concentration-dependent insecticidal activity against both tephritid fruit flies (*p* < 0.05 for all ANOVAs). N-hexane was the most potent, followed by methanol, then ethyl acetate. *B. cucurbitae* was generally more susceptible than *B. zonata*, although the difference diminished at higher concentrations and longer exposures, especially with n-hexane.

### 3.5. Analysis of R. communis Extracts Using Fourier Transform Infrared Spectroscopy (FTIR)

**Figure 2:**
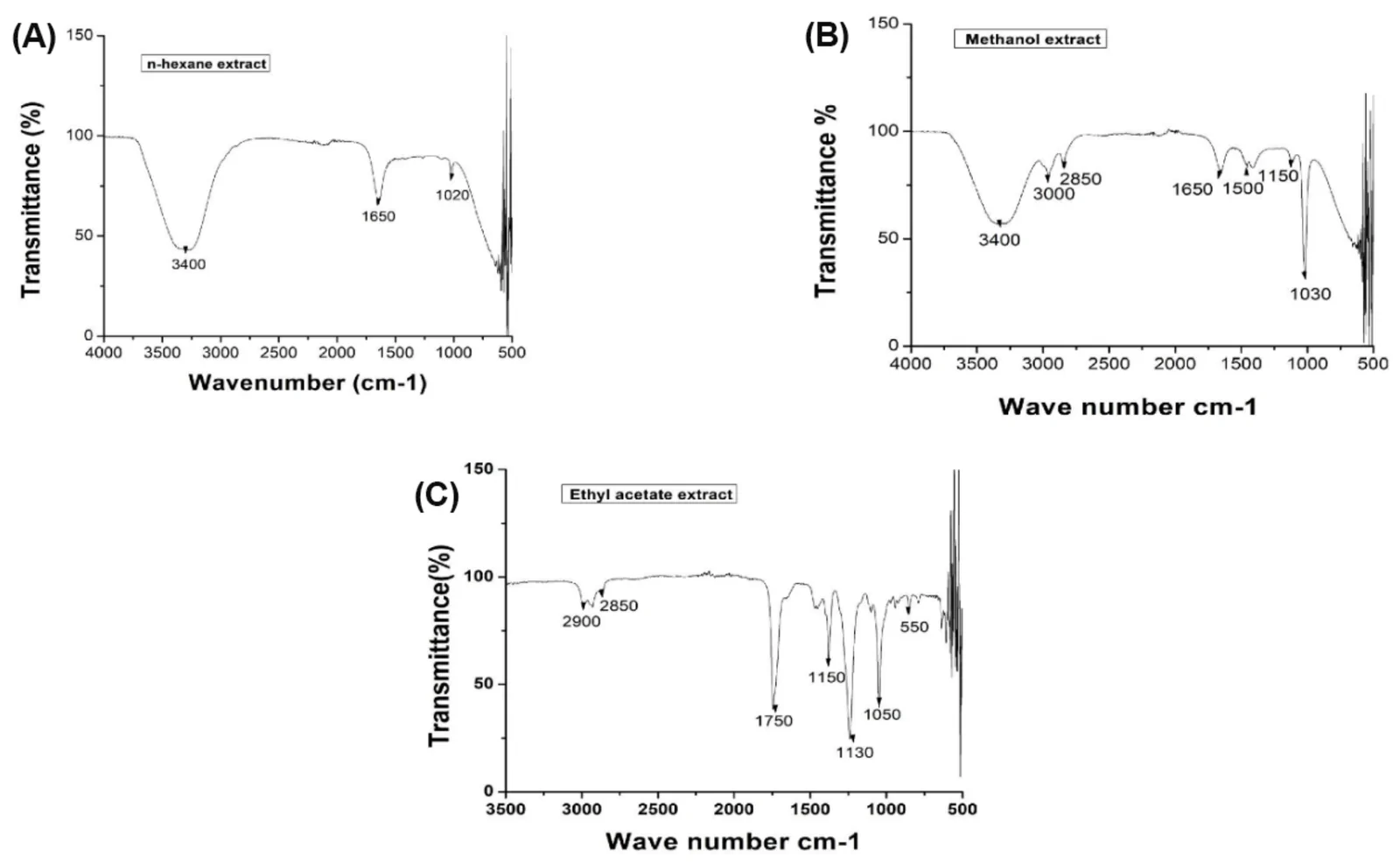
FTIR spectra of different extracts of *R. communis* leaves. (**A**) *n*-hexane, (**B**) methanol extract and (**C**) ethyl acetate.

FTIR spectroscopy of the extracts (Figure 2) showed several characteristic absorption bands corresponding to different functional groups. A broad absorption band around 3400 cm^−1^ was assigned to O–H stretching vibrations, suggesting the presence of hydroxyl-containing compounds such as alcohols or phenolics. The absorption bands near 1650 cm^−1^ were tentatively assigned to C=C bending vibrations of aromatic or unsaturated compounds, and or amide-related vibrations, suggesting the possible presence of nitrogen-containing or unsaturated constituents. Peaks around 1020–1150 cm^−1^ were tentatively assigned to C–F stretching vibrations, suggesting the possible presence of aliphatic fluoro-related functional groups. In the methanol extract, absorption bands near 3000 cm^−1^ and 2850 cm^−1^ corresponded to O–H and C–H stretching vibrations, respectively, indicating alcohols and alkanes. Similarly, in the ethyl acetate extract, peaks near 2900 and 2800 cm^−1^ suggested alkane-related C–H stretching, while the band at 1750 cm^−1^ was attributed to ester carbonyl (C=O) stretching vibrations.

Another peaks were observed at 781.17, 875.68, 923.90, 1018.41, 1240.23, 1319.31, 1361.74, 2357.01, 2850.79, and 2920.23 cm^−1^ (Table 4), further indicating the chemical complexity of the extracts. Overall, FTIR analysis provided qualitative information regarding the major functional groups present in the extracts of *R. communis*. However, FTIR spectroscopy alone cannot conclusively identify specific compounds or determine their biological activity. Therefore, the observed absorption bands should be interpreted as indicative of general chemical functionalities rather than definitive evidence of particular phytochemicals or insecticidal modes of action. FTIR analysis provides qualitative information on functional groups only. It cannot identify specific compounds or determine biological activity.

### 3.6. GC-MS (Gas Chromatography–Mass Spectrometry) Analysis of Extract of R. communis

**Figure 3:**
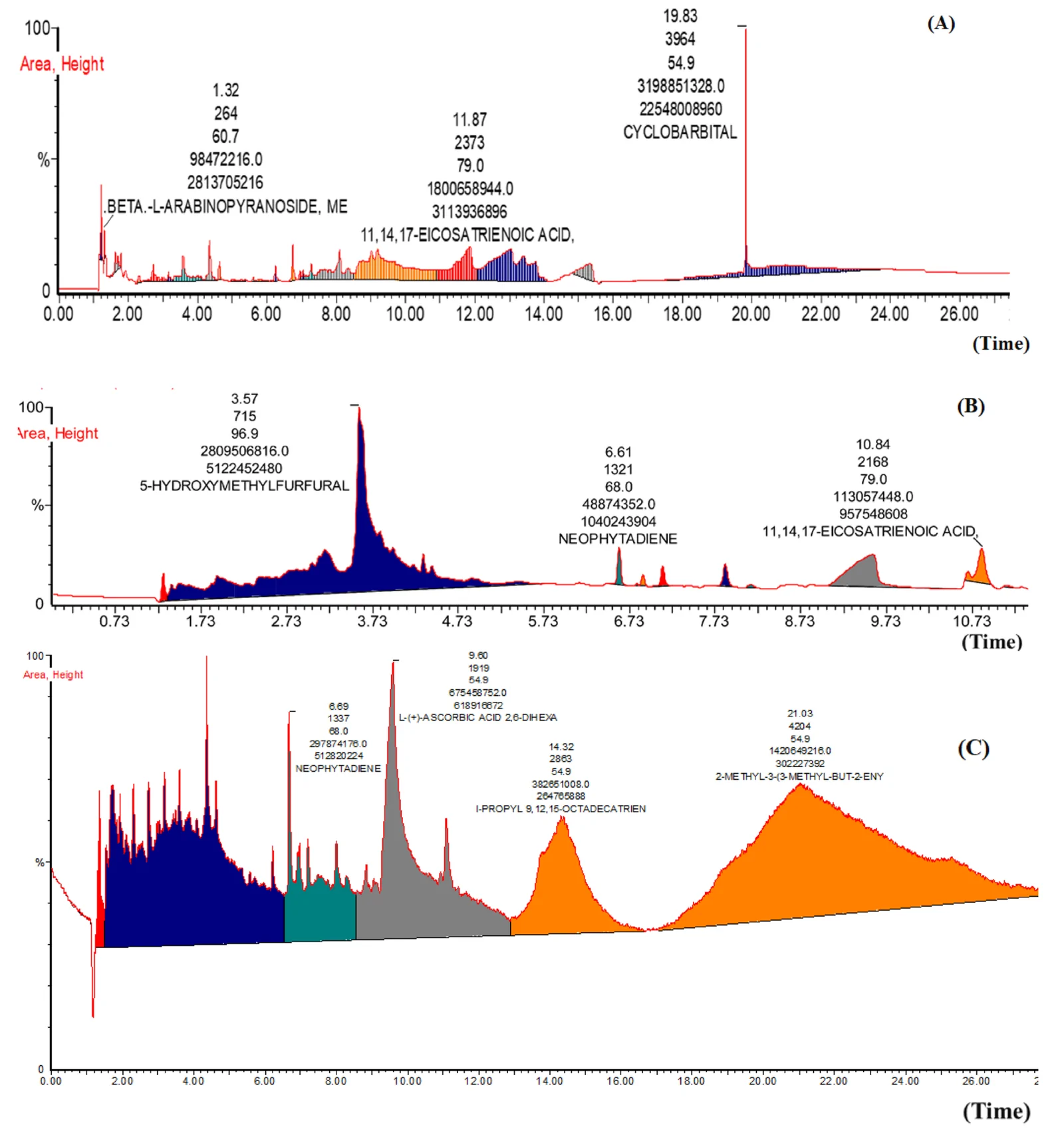
(**A**) GC–MS chromatogram of the methanol extract; (**B**) GC–MS chromatogram of the n-hexane extract; (**C**) GC–MS chromatogram of the ethyl acetate extract. Chromatographic profiles were obtained from the previously reported phytochemical characterization study (Akbar et al. [10]) and are presented as complementary chemical information. Compound assignments were based on GC–MS spectral library matching and should be regarded as tentative, as authentic standards, retention indices, and independent structural confirmation were not available. The extracts analyzed by GC–MS were independently prepared and were not identical to the ethanol crude extracts used in the insect bioassays of the present study.

GC–MS analysis was used to characterize the chemical constituents of independently prepared solvent extracts of *R. communis* leaves. The chromatographic profiles presented in Figure 3 were obtained from our previous phytochemical characterization study (Akbar et al. [11]) and are included here to provide qualitative information on the chemical composition of *R. communis* extracts. Importantly, the extracts subjected to GC–MS analysis were independently prepared solvent-specific extracts and were not identical to the ethanol crude extracts used in the insect bioassays. Therefore, the GC–MS data are presented as complementary phytochemical information and should not be interpreted as a direct chemical characterization of the bioassay materials. The detailed compound identification and chemical profiling were previously reported, whereas the present study primarily evaluates the comparative insecticidal activity of *R. communis* extracts against *B. zonata* and *B. cucurbitae*.

#### 3.6.1. Methanolic Extract

The methanolic extract showed multiple chromatographic peaks corresponding to different phytochemical constituents (Figure 3, Table 5). Several compounds were tentatively assigned through GC–MS library matching, including 5-hydroxymethylfurfural (RT 3.57 min), neophytadiene (RT 6.61–6.69 min), and 11,14,17-eicosatrienoic acid (RT 10.84 min). Other detected constituents included L-(+)-ascorbic acid 2,6-dihexadecanoate, 1-propyl 9,12,15-octadecatrienate, and other hydrocarbon derivatives. Because authentic reference standards, retention indices, and independent structural confirmation were not available, these compound assignments should be regarded as tentative rather than confirmed identifications.

#### 3.6.2. n-Hexane Extract

The n-hexane extract showed several chromatographic peaks corresponding to distinct non-polar phytochemical constituents (Figure 3, Table 5). Major compounds were tentatively assigned by library matching, including neophytadiene, a fatty acid ester derivative, and a long-chain unsaturated hydrocarbon derivative. Compound assignments were based on comparison with the NIST spectral database and should be considered tentative because authentic analytical standards, retention indices, and independent structural confirmation were not available.

#### 3.6.3. Ethyl Acetate Extract

The ethyl acetate extract exhibited several chromatographic peaks (Figure 3, Table 5). Among the tentatively assigned constituents were β-L-arabinopyranoside, 11,14,17-eicosatrienoic acid, and cyclobarbital. The library match corresponding to cyclobarbital was considered particularly uncertain and was therefore treated only as a tentative spectral assignment. Because authentic reference standards, retention indices, and independent structural confirmation were not available, this assignment cannot be considered a confirmed constituent of *R. communis* and should not be interpreted as evidence of biological activity.

### 3.7. Multivariate Analysis

**Figure 4:**
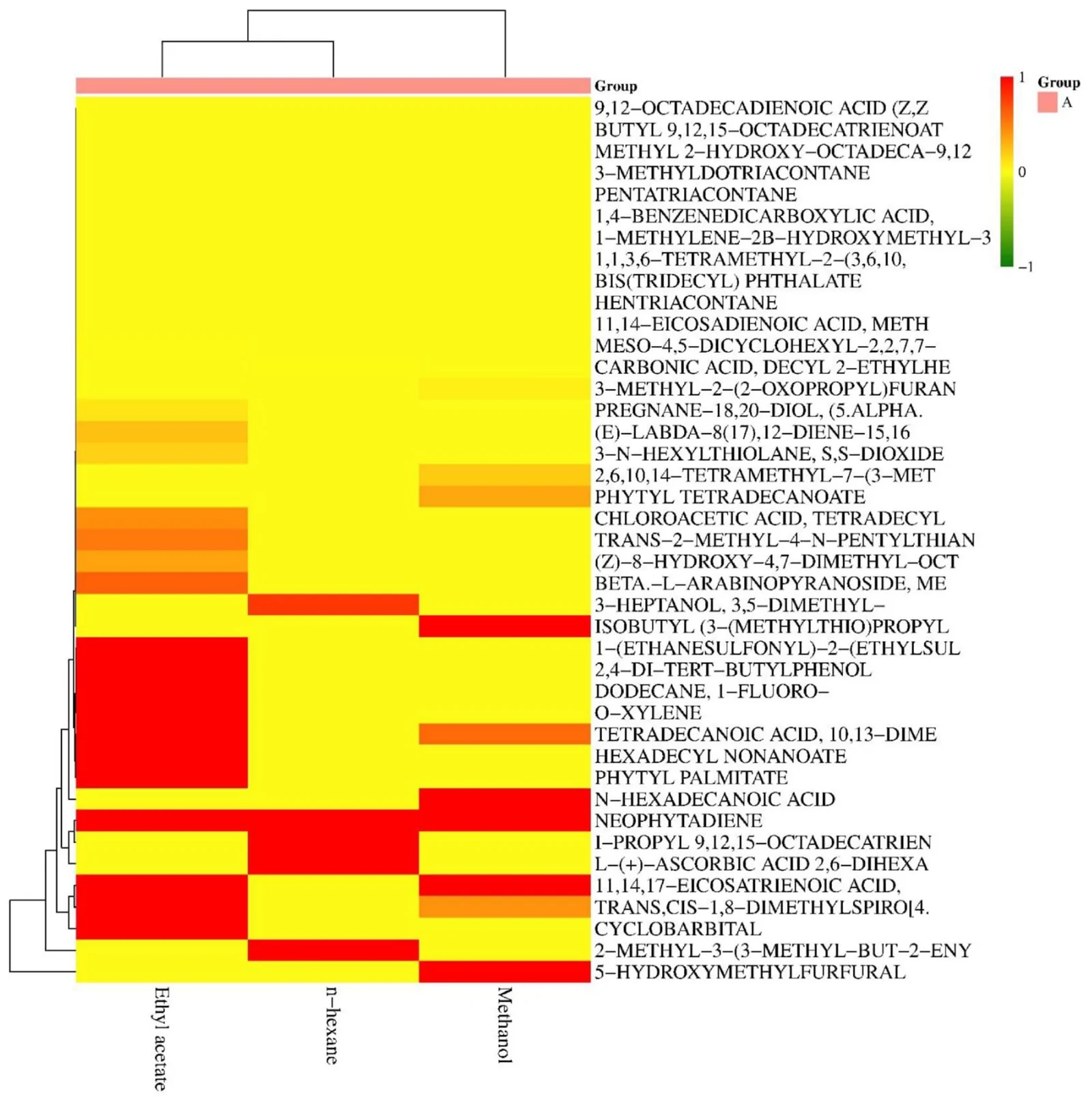
Hierarchical clustering of tentatively identified compounds detected in independently prepared methanol, n-hexane, and ethyl acetate extracts of *R. communis* leaves.

Hierarchical cluster analysis (HCA; Figure 4) showed differences in the chemical profiles of the independently prepared methanol, n-hexane, and ethyl acetate extracts. The distribution of detected compounds indicated greater chemical complexity in the methanol and ethyl acetate extracts than in the n-hexane extract. The detailed list of compounds detected in each extract and their relative abundances is presented in Table 5. Neophytadiene was detected across all three solvent extracts, whereas several other constituents were detected in only one or two extracts. For example, 11,14,17-eicosatrienoic acid and trans-cis-1,8-dimethylspiro [4] were detected in the methanol and ethyl acetate extracts, while other constituents showed more restricted distributions. HCA further revealed clustering patterns among the detected compounds and solvent extracts, reflecting similarities and differences in their chemical profiles.

Compounds including hentriacontane and 3-methyl-2-(2-oxopropyl)furan were detected across multiple solvent extracts, whereas tetradecanoic acid 10,13-dimethyl derivative was detected in the methanol and ethyl acetate extracts (Table 6). These findings demonstrate that solvent extraction conditions influenced the qualitative chemical composition of *R. communis* leaf extracts.

Importantly, the chemical profiles shown in Figure 4 and Table 5 and Table 6 were obtained from independently prepared solvent-specific extracts and should therefore not be interpreted as direct chemical characterization of the ethanol crude extracts used in the insect bioassays. Accordingly, the present HCA results provide complementary information on the phytochemical composition of *R. communis* extracts but do not establish a causal relationship between individual GC–MS-identified compounds and insect mortality. The contribution of individual compounds to the observed insecticidal activity requires confirmation using chemically matched extracts, purified compounds, and compound-specific bioassays.

## 4. Discussion

Medicinal plants have long been recognized as reservoirs of bioactive secondary metabolites with insecticidal, antimicrobial, anti-inflammatory and antioxidant properties [19,40]. Among these plant species, *R. communis* (castor bean) has attracted considerable interest due to its diverse phytochemical composition and biological properties [26]. The present study investigated the insecticidal efficacy of sequentially extracted extracts (methanol, n-hexane, ethyl acetate) from *R. communis* leaves against two major tephritid pests, *B. cucurbitae* and *B. zonata*. Using a combination of bioassays, GC–MS, and FTIR analyses, we demonstrated that solvent polarity strongly influences the chemical composition of *R. communis* leaf extract. It also significantly affects the resulting toxicity. 11,14,17-eicosatrienoic acid was among the compounds tentatively detected in the independently prepared extracts and has previously been reported in *R. communis* and other plant extracts with potential biological activity [10]. However, its contribution to the insecticidal activity observed in the present study remains unverified. The current study extends this work by providing new comparative data for *B. cucurbitae*. The present study comprised two complementary but analytically distinct approaches: insect bioassays using ethanol crude extracts diluted with different solvents, and chemical characterization of independently prepared solvent-specific extracts. The GC–MS profiles therefore provide contextual information on the phytochemical composition of *R. communis* leaves but do not directly characterize the chemical composition of the materials used in the bioassays. Consequently, the present data cannot establish a direct relationship between individual GC–MS-identified compounds and the mortality responses observed in the bioassays. Any potential contribution of the reported compounds to insecticidal activity should therefore be considered hypothetical until confirmed using chemically matched extracts, bioassay-guided fractionation, purified compounds, and appropriate dose–response experiments.

Across all three solvents (methanol, n-hexane, and ethyl acetate), the mortality of both flies increased significantly with increasing concentration and exposure time. One-way ANOVA confirmed significant concentration effects for each solvent–insect–exposure time combination (*p* < 0.05). These findings showed a consistent concentration-dependent mortality response. Such time- and concentration-dependent toxicity is commonly reported for plant-derived insecticides. Plant extracts often exert insecticidal activity through multiple sublethal mechanisms, including antifeedant effects, growth disruption, and neurotoxicity, which may ultimately contribute to mortality [41,42]. The n-hexane extract exhibited the highest insecticidal activity. Complete (100%) mortality was achieved within a 48 h at a 2% concentration, while near complete control (96.7–100%) in *B. cucurbitae* and near complete control (96.7–100%) of both species were observed after 72 h. These outcomes suggest that some highly active constituents of *R. communis* may be relatively non-polar and moderately lipophilic and are efficiently recovered in the n-hexane extract. The methanol extracts also performed substantial insecticidal activity and prolonged the exposure duration. Mortality of *B. cucurbitae* reached 96.7% after 72 h at the 2% concentration, exhibiting the presence of more polar active compounds. Ethyl acetate extracts were moderately less effective, although they still caused significant mortality of 86.7–93.3% at the 2% concentration after 72 h. The differential activity across solvents points to a complex phytochemical matrix. The differential activity among solvent extracts may be attributed to differences in their chemical composition, as solvents with varying polarity can selectively extract different groups of bioactive constituents, including terpenoids and fatty acid derivatives identified through GC–MS analysis [18,43].

Species-specific differences in susceptibility were observed among the tested extracts. Based on observed mortality at equivalent concentrations, *B. cucurbitae* frequently showed higher mortality than *B. zonata*, particularly for methanol and n-hexane extracts. Direct statistical comparisons of LC_50_ and LC_90_ values between species were not performed because separate probit analyses were conducted for each species. Therefore, differences in lethal concentration estimates were interpreted based on the magnitude of LC values and their associated 95% confidence intervals. The observed mortality at individual concentrations and the estimated lethal concentrations represent different aspects of toxicity; mortality reflects the response at specific tested doses, whereas LC values describe the fitted concentration–mortality relationship across all tested concentrations. Although similar mortality percentages were observed between *B. zonata* and *B. cucurbitae* at certain ethyl acetate concentrations, the estimated LC_90_ values differed because these parameters were derived from the overall concentration mortality regression model rather than from mortality at a single concentration. The LC_90_ estimate is influenced by the slope of the probit regression and the variability of responses across all tested concentrations. Therefore, species exhibiting comparable mortality at tested doses may still produce different LC_90_ estimates due to differences in their fitted dose–response relationships. In the present study, separate probit analyses were conducted for each species; therefore, differences in LC_50_ and LC_90_ values were interpreted based on estimated values and their 95% confidence intervals, and not as statistically significant differences between species. This interpretation applies to all three solvent preparations (methanol, ethyl acetate, and n-hexane). An interesting interspecific variation in susceptibility was observed between the two species. For instance, at the 2% concentration, mortality reached 93.0% in *B. cucurbitae* compared with 86.7% in *B. zonata* after 48 h exposure. In contrast, the ethyl acetate extract showed relatively similar toxicity patterns between species, although *B. cucurbitae* exhibited higher mortality at the highest concentration after 72 h (83.33%) compared with *B. zonata* (66.67%). This suggest that some compounds recovered in the ethyl acetate extract may exhibit relatively greater toxicity to *B. zonata.* Such differences may be associated with species-specific physiological or biochemical responses; however, the mechanisms responsible require further investigation [44,45]. The n-hexane and methanol fractions were highly effective against both species of fruit flies under practical considerations. This findings highlight that *R. communis*-based extracts have potential for managing mixed populations of these tephritid pests; however, field validation is required before practical application.

GC–MS analysis of the independently prepared solvent extracts revealed several tentatively identified constituents, including neophytadiene, L-(+)-ascorbic acid 2,6-dihexadecanoate, 1-propyl 9,12,15-octadecatrienoate, 11,14,17-eicosatrienoic acid, and other compounds. Previous studies have reported diverse phytochemical groups in *R. communis*, including alkaloids, flavonoids, terpenoids, tannins, and glycosides [46]. Some compounds detected in *R. communis* extracts have previously been associated with biological activities, including potential insecticidal, repellent, antioxidant, or antimicrobial effects [47,48]. However, these previous reports cannot be directly used to attribute the insecticidal activity observed in the present study to any individual compound.

Importantly, the GC–MS analyses in the present study were conducted using independently prepared solvent-specific extracts, whereas the insect bioassays were performed using ethanol crude extracts diluted with different solvents. Therefore, the detected compounds and their relative abundances cannot be assumed to represent the chemical composition of the bioassay materials. Moreover, the GC–MS identifications were based primarily on library matching without confirmation using authentic analytical standards. Thus, the detected constituents should be regarded as tentative chemical markers rather than confirmed active ingredients. The present findings provide a basis for future investigation but do not establish a direct causal relationship between individual phytochemicals and insect mortality.

FTIR analysis qualitatively confirmed the presence of multiple functional groups. These consist of aliphatic alcohols, esters, alkanes, and aliphatic amines [49]. This exhibits a diverse array of complex chemicals. Although, FTIR only provides confirmation of functional groups. It does not provide definitive molecular structures. It also does not establish a direct correlation between specific spectral bands and biological activity. GC-MS identifications were based on library matching without analytical standards. Therefore, all chemical assignments should be considered tentative. FTIR data confirms functional groups only, not molecular structures or biological activity. It is supplementary to GC-MS results. Therefore, the FTIR data should be regarded as supplementary to the more conclusive GC–MS results.

A major limitation of the present study is that the chemical characterization and insect bioassays were conducted using different extract preparations. The bioassays employed ethanol crude extracts diluted with different solvents, whereas the GC–MS analyses were performed on independently prepared methanol, n-hexane, and ethyl acetate extracts. Consequently, the compounds detected by GC–MS cannot be directly linked to the mortality responses observed in the bioassays. The present study therefore does not provide experimental evidence that any particular compound, including 11,14,17-eicosatrienoic acid or neophytadiene, was responsible for the observed insecticidal activity.

Furthermore, the bioassays were conducted using crude mixtures rather than purified compounds, and GC–MS compound assignments were based on library matching without confirmation using authentic standards. Therefore, the reported constituents should be considered potential candidates for future investigation rather than confirmed active principles. The observed insecticidal activity may result from the combined effects of multiple constituents; however, synergistic or additive interactions were not experimentally evaluated in the present study. Future research should employ chemically matched bioassay extracts followed by bioassay-guided fractionation, purification of candidate compounds, structural confirmation using authentic reference standards, and individual and combination bioassays to determine their actual contribution to insecticidal activity.

Another limitation of the present study relates to the estimation of lethal concentration values through probit analysis. Although probit analysis is widely applied for determining LC_50_ and LC_90_ values in insect toxicology, the accuracy of these estimates depends on the number and distribution of tested concentrations and the availability of a broad mortality response range. In the present study, four concentrations (0.5, 1.0, 1.5, and 2.0%) were selected based on preliminary toxicity screening and produced clear concentration-dependent mortality patterns. However, the limited number of concentrations may have reduced the precision of some lethal concentration estimates, as reflected by wider confidence intervals observed for certain treatments, particularly at shorter exposure periods. Therefore, these LC_50_ and LC_90_ values should be interpreted cautiously. Future studies involving pure-compound bioassays will be necessary to confirm the contribution of individual compounds and to further elucidate their potential modes of action.

The strong insecticidal activity observed against *B. cucurbitae* and *B. zonata* in the present study parallels findings reported for other plant species, such as *Cascabela peruviana,* whose ethanol extracts caused larval mortality and developmental disruption in *Drosophila melanogaster* [50]. Similarly, insecticidal activity has also been reported for *R. communis* against *Spodoptera cosmioides*, *S. frugiperda* [18], *Musca domestica* [51] and mosquito larvae [52]. Collectively, these studies suggest that *R. communis* phytochemicals can interfere with hormonal regulation, disrupt normal growth and metamorphosis, and exert potent larvicidal and ovicidal effects. The present study extends this knowledge to two economically important tephritid fruit flies, providing a strong foundation for further development of *R. communis*-based botanical insecticides.

The observation that n-hexane extract achieved 100% mortality at a 2% concentration within 48 h, and that even a 1.5% concentration gave >90% mortality after 72 h, suggests that field-applicable concentrations may be feasible. Given the biodegradable nature of plant extracts, they represent promising alternatives to synthetic insecticides, particularly for integrated pest management (IPM) in vegetable and fruit crops. However, before large-scale application can be recommended, further studies are needed to evaluate: (i) non-target effects on pollinators, natural enemies, and soil organisms; (ii) residual activity and photostability under field condition; (iii) formulation development to enhance field persistence and stability; and (iv) mammalian toxicity; although many detected compounds possess reported anticancer, antibacterial or wound-healing properties, their toxicological safety profiles must be established before practical application.

We acknowledge that our study focused only on adult contact toxicity. Future studies should evaluate the effects of *R. communis* extracts on oviposition deterrence, larval and pupal mortality, and adult behavioral responses such as feeding and mating, as these parameters are also relevant for comprehensive fruit fly management.

## 5. Conclusions

*Ricinus communis* leaf extracts exhibited significant insecticidal activity against *B. cucurbitae* and *B. zonata*, with the n-hexane extract showing the highest efficacy and causing 100% mortality of *B. cucurbitae* at a 2% concentration within 48 h. GC–MS analysis identified several major constituents, including neophytadiene and fatty acid derivatives, which may contribute to the observed insecticidal activity. FTIR analysis further confirmed the presence of diverse functional groups in the extracts. These findings highlight the potential of *R. communis* extracts as promising botanical insecticides; however, further studies involving compound isolation, mode-of-action investigations, safety assessment, and field validation are required before practical application.

## Figures and Tables

**Table 1:** Methanol concentration-dependent mortality and probit analysis for *B. zonata* and *B. cucurbitae* after 24, 48 and 72 h exposure.

Time	Conc. (%)	Corrected Mortality (%) Mean ± SD		Df	Toxicity Parameters (95% CI)		
		*B. zonata*	*B. cucurbitae*			*B. zonata*	*B. cucurbitae*
24 h	0.5	6.67 ± 5.77 b	20.00 ± 0.00 b	2	LC_50_ (%)	1.12 (0.04–1.78)	5.72 (3.29–234.15)
	1.0	13.33 ± 5.77 ab	26.67 ± 5.77 b	2	LC_90_ (%)	35.56 (1.10–1150)	14.96 (1.67–133.7)
	1.5	16.67 ± 2.89 ab	33.33 ± 5.77 ab	2	Slope ± SE	4.60 ± 0.44	1.20 ± 0.44
	2.0	23.33 ± 2.89 a	46.67 ± 5.77 a	2	F	3.80	3.89
	Control	0.00 ± 0.00 ns	0.00 ± 0.00 ns	2	*P*	0.0600	0.0553
	LSD (conc.)	11.00	18.83		χ^2^	3.80	0.56
48 h	0.5	13.33 ± 5.77 b	30.00 ± 5.77 c	2	LC_50_ (%)	0.50 (0.00–1.15)	2.94 (1.64–4.26)
	1.0	16.67 ± 5.77 b	40.00 ± 0.00 bc	2	LC_90_ (%)	1.20 (0.00–1.93)	14.06 (1.81–109.4)
	1.5	20.00 ± 5.77 ab	43.33 ± 2.89 b	2	Slope ± SE	3.40 ± 0.38	1.05 ± 0.38
	2.0	30.00 ± 5.77 a	56.67 ± 7.64 a	2	F	4.03	8.24
	Control	0.00 ± 0.00 ns	0.00 ± 0.00 ns	2	*P*	0.0400	0.0079
	LSD (conc.)	10.17	13.38		χ^2^	4.03	8.24
72 h	0.5	20.00 ± 0.00 b	30.77 ± 0.00 b	2	LC_50_ (%)	0.30 (0.00–0.78)	1.38 (0.00–2.27)
	1.0	23.33 ± 2.89 b	38.46 ± 6.66 b	2	LC_90_ (%)	0.66 (0.00–1.25)	22.80 (6.82–26.91)
	1.5	30.00 ± 5.77 ab	46.15 ± 6.66 ab	2	Slope ± SE	3.74 ± 0.35	1.05 ± 0.35
	2.0	43.33 ± 7.64 a	61.54 ± 7.64 a	2	F	5.05	4.00
	Control	0.00 ± 0.00 ns	0.00 ± 0.00 ns	2	*P*	0.0300	0.0519
	LSD (conc.)	13.66	20.70		χ^2^	5.05	4.04

LC_50_ and LC_90_ = model-estimated concentrations (%) associated with 50% and 90% mortality, respectively; CI = 95% confidence interval; SE = standard error; F = F-statistic from one-way ANOVA testing the effect of concentration; P = probability value; χ^2^ = chi-square statistic for the probit goodness-of-fit test; df = degrees of freedom; ns = not significant (*p* > 0.05); LSD = least significant difference for post hoc mean comparisons (α = 0.05). Different lowercase letters indicate that the means are significantly different from each other at *p* = 0.05. LC_50_ and LC_90_ estimates outside the tested concentration range (0.5–2.0%) represent extrapolated model estimates and should therefore be interpreted cautiously. Estimates substantially beyond the tested range, particularly those accompanied by wide confidence intervals, were not considered experimentally validated concentrations. The χ^2^ goodness-of-fit test was used to evaluate the fit of the probit model to the concentration–mortality data.

**Table 2:** N-hexane concentration-dependent mortality and probit analysis for *B. zonata* and *B. cucurbitae* after 24, 48 and 72 h exposure.

Time	Conc. (%)	Corrected Mortality (%) Mean ± SD		Df	Toxicity Parameters (95% CI)		
		*B. zonata*	*B. cucurbitae*			*B. zonata*	*B. cucurbitae*
24 h	0.5	20.00 ± 5.77 b	13.33 ± 5.77 b	2	LC_50_ (%)	2.18 (1.80–2.70)	2.04 (1.60–2.50)
	1.0	27.00 ± 10.00 b	20.00 ± 10.00 b	2	LC_90_ (%)	20.70 (14.50–29.50)	8.90 (6.20–12.80)
	1.5	34.00 ± 0.00 ab	40.00 ± 0.00 a	2	Slope ± SE	1.20 ± 0.45	2.00 ± 0.45
	2.0	48.00 ± 5.77 a	53.33 ± 5.77 a	2	F	3.89	12.00
	Control	0.00 ± 0.00 ns	0.00 ± 0.00 ns	2	*P*	0.055	0.000
	LSD (conc.)	18.83	18.83		χ^2^	1.25	0.89
48 h	0.5	40.00 ± 11.55 b	39.00 ± 11.55 b	2	LC_50_ (%)	1.22 (1.00–1.50)	0.67 (0.50–0.90)
	1.0	47.00 ± 5.77 ab	59.00 ± 5.77 b	2	LC_90_ (%)	15.60 (10.80–22.50)	1.81 (1.40–2.30)
	1.5	53.00 ± 10.00 a	86.00 ± 10.00 a	2	Slope ± SE	1.04 ± 0.34	3.00 ± 0.34
	2.0	67.00 ± 5.00 a	93.00 ± 5.00 a	2	F	3.77	31.10
	Control	00.00 ± 0.00 ns	00.00 ± 0.00 ns	2	*P*	0.059	0.000
	LSD (conc.)	19.19	12.75		χ^2^	0.78	1.20
72 h	0.5	63.33 ± 5.77 b	78.67 ± 10.26 b	2	LC_50_ (%)	0.65 (0.55–0.77)	0.33 (0.25–0.42)
	1.0	73.33 ± 11.55 ab	90.00 ± 10.00 ab	2	LC_90_ (%)	2.48 (2.00–3.10)	1.01 (0.80–1.30)
	1.5	76.67 ± 5.77 ab	93.33 ± 5.77 a	2	Slope ± SE	1.50 ± 0.31	2.60 ± 0.31
	2.0	90.00 ± 10.00 a	100.00 ± 0.00 a	2	F	4.33	7.00
	Control	13.33 ± 11.55 ns	15.00 ± 11.55 ns	2	*P*	0.043	0.020
	LSD (conc.)	21.96	15.04		χ^2^	0.56	0.92

LC_50_ and LC_90_ = model-estimated concentrations (%) associated with 50% and 90% mortality, respectively; CI = 95% confidence interval; SE = standard error; F = F-statistic from one-way ANOVA testing the effect of concentration; P = probability value; χ^2^ = chi-square statistic for the probit goodness-of-fit test; df = degrees of freedom; ns = not significant (*p* > 0.05); LSD = least significant difference for post hoc mean comparisons (α = 0.05). Different lowercase letters indicate that the means are significantly different from each other at *p* = 0.05. LC_50_ and LC_90_ estimates outside the tested concentration range (0.5–2.0%) represent extrapolated model estimates and should therefore be interpreted cautiously. Estimates substantially beyond the tested range, particularly those accompanied by wide confidence intervals, were not considered experimentally validated concentrations. The χ^2^ goodness-of-fit test was used to evaluate the fit of the probit model to the concentration–mortality data.

**Table 3:** Ethyl-acetate concentration-dependent mortality and probit analysis for *B. zonata* and *B. cucurbitae* after 24, 48 and 72 h exposure.

Time	Conc. (%)	Corrected Mortality (%) Mean ± SD		Df	Toxicity Parameters (95% CI)		
		*B. zonata*	*B. cucurbitae*			*B. zonata*	*B. cucurbitae*
24 h	0.5	20.00 ± 0.00 b	13.33 ± 11.55 b	2	LC_50_	3.02 (2.50–3.70)	2.55 (2.10–3.10)
	1.0	26.67 ± 11.55 b	20.00 ± 0.00 ab	2	LC_90_	40.10 (28.50–56.30)	15.70 (11.20–22.00)
	1.5	33.33 ± 11.55 ab	33.33 ± 11.55 ab	2	Slope ± SE	1.14 ± 0.45	1.52 ± 0.45
	2.0	46.67 ± 11.55 a	46.67 ± 23.09 a	2	F	3.89	12.00
	Control	0.00 ± 0.00 ns	0.00 ± 0.00 ns	2	*P*	0.055	0.008
	LSD (conc.)	18.83	25.45		χ^2^	0.45	0.89
48 h	0.5	30.83 ± 15.77 c	21.67 ± 2.89 c	2	LC_50_	1.83 (1.50–2.20)	1.61 (1.30–1.90)
	1.0	38.33 ± 12.58 bc	35.00 ± 8.66 bc	2	LC_90_	24.30 (17.60–33.50)	5.87 (4.50–7.60)
	1.5	43.33 ± 5.77 b	41.67 ± 17.56 ab	2	Slope ± SE	1.04 ± 0.34	1.97 ± 0.34
	2.0	56.67 ± 5.77 a	56.67 ± 5.77 a	2	F	3.77	31.10
	Control	0.00 ± 0.00 ns	0.00 ± 0.00 ns	2	*P*	0.0593	0.000
	LSD (conc.)	13.38	15.60		χ^2^	0.65	1.20
72 h	0.5	37.50 ± 12.50 b	33.33 ± 14.43 b	2	LC_50_	1.28 (1.00–1.60)	0.92 (0.70–1.20)
	1.0	50.00 ± 25.00 b	50.00 ± 0.00 ab	2	LC_90_	7.32 (5.50–9.80)	3.18 (2.40–4.20)
	1.5	54.17 ± 7.22 ab	66.67 ± 14.43 a	2	Slope ± SE	1.42 ± 0.31	1.71 ± 0.31
	2.0	66.67 ± 28.87 a	83.33 ± 14.43 a	2	F	4.33	7.00
	Control	0.00 ± 0.00 ns	00.00 ± 0.00 ns	2	*P*	0.748	0.630
	LSD (conc.)	20.70	14.04		χ^2^	0.58	0.92

LC_50_ and LC_90_ = model-estimated concentrations (%) associated with 50% and 90% mortality, respectively; CI = 95% confidence interval; SE = standard error; F = F-statistic from one-way ANOVA testing the effect of concentration; P = probability value; χ^2^ = chi-square statistic for the probit goodness-of-fit test; df = degrees of freedom; ns = not significant (*p* > 0.05); LSD = least significant difference for post hoc mean comparisons (α = 0.05). Different lowercase letters indicate that the means are significantly different from each other at *p* = 0.05. LC_50_ and LC_90_ estimates outside the tested concentration range (0.5–2.0%) represent extrapolated model estimates and should therefore be interpreted cautiously. Estimates substantially beyond the tested range, particularly those accompanied by wide confidence intervals, were not considered experimentally validated concentrations. The χ^2^ goodness-of-fit test was used to evaluate the fit of the probit model to the concentration–mortality data.

**Table 4:** FTIR peaks observed in extracts ion in selected extracts of leaves of *R. communis*.

Wavenumber (cm^−1^)	Bond	Functional Group
3400	O-H	Hydrogen bonded Alcohol
3000	O-H	Alcohol
2900	C-H	Alkane
2850	C-H	Alkane
1750	C=O	Ester
1650	N-H	Secondary amine
1500	N-O	Nitro compounds
1150	C-F	Aliphatic flouro compounds
1130	C-F	Aliphatic flouro compounds
1030	C-F	Aliphatic flouro compounds
1020	C-F	Aliphatic flouro compounds
550	C-Br	Halogen compound

**Table 5:** Tentatively identified chemical constituents of independently prepared *R. communis* leaf extracts based on GC–MS library matching.

Extract	Compound Identified	Chemical Class	Retention Time (min)	Relative Abundance (%)	Literature-Reported Bioactivity
Methanol	5-Hydroxymethylfurfural	Furan derivative	3.57	81.78	Antioxidant; intermediate metabolite; antimicrobial potential [33]
Methanol	Neophytadiene	Diterpenoid hydrocarbon	6.61	1.42	Plant defense metabolite; antimicrobial activity reported [34]
Methanol	11,14,17-Eicosatrienoic acid	Polyunsaturated fatty acid	10.84	3.29	Anti-inflammatory lipid; bioactive fatty acid [35]
n-Hexane	Neophytadiene	Diterpenoid hydrocarbon	6.69	7.53	Antioxidant-associated terpenoid; stress-response compound [34]
n-Hexane	Fatty acid ester derivative (putative)	Lipid derivative	9.60	7.08	Lipid-based bioactive molecules [36]
n-Hexane	Long-chain unsaturated hydrocarbon derivative	Polyunsaturated lipid	14.32	9.67	Membrane-associated lipid; metabolic intermediate [37]
Ethyl acetate	Beta-L-arabinopyranoside	Glycoside	1.32	0.63	Plant glycoside; antioxidant potential reported [37]
Ethyl acetate	11,14,17-Eicosatrienoic acid	Fatty acid	11.85	11.52	Omega-type fatty acid; anti-inflammatory lipid [38]
Ethyl acetate	Cyclobarbital (tentative identification)	Barbiturate-like structure	19.81	20.46	CNS-active compound class (possible contaminant/artefact) [39]

Note: Compound identities were assigned tentatively based on mass spectral library matching. Because authentic analytical standards, retention indices, and independent structural confirmation were not available, the reported compound assignments should not be considered definitive. Relative abundances represent the GC–MS profiles of independently prepared solvent-specific extracts and should not be interpreted as direct chemical characterization of the ethanol crude extracts used in the insect bioassays.

**Table 6:** Exploratory associations between selected tentatively identified GC–MS compounds and mortality responses of *B. cucurbitae* and *B. zonata*.

Compounds	*B. cucurbitae*		*B. zonata*	
	R-Square	*P*-Value	R-Square	*P*-Value
Neophytadiene	0.20	0.004	0.21	0.005
11,14,17-Eicosatrienoic Acid	0.61 **	0.000	0.64 **	0.000
3-Methyl-2-(2-oxopropyl)Furan	0.03	0.261	0.04	0.264
Hentriacontane	0.03	0.229	0.04	0.232
*Trans-cis*, 1,8-Dimethylspiro [4]	0.58 **	0.000	0.60 **	0.000
Tetradecanoic acid 10,13-Dime	0.21 *	0.015	0.23 *	0.017

R^2^ = coefficient of determination; P = probability value. The analyses were exploratory and evaluated statistical associations between the available compound-abundance and mortality data. The tentatively identified GC–MS compounds were obtained from independently prepared solvent-specific extracts and were not chemically identical to the ethanol crude extracts used in the insect bioassays. Therefore, the observed associations represent statistical relationships only and do not establish causation or demonstrate the insecticidal activity of individual compounds. No compound-specific LC_50_ values were experimentally determined in the present study. Compound identities were assigned tentatively based on GC–MS spectral library matching and were not confirmed using authentic analytical standards, retention indices, or independent structural characterization. *p* < 0.05 (*) and *p* < 0.01 (**).

## Data Availability

Research data are only available upon request for corresponding authors.

## Supplementary Materials

The following supporting information can be downloaded at https://www.mdpi.com/article/10.3390/insects17080811/s1, Table S1: Treatment combinations, concentrations, and experimental design used for evaluation of the contact toxicity of crude ethanol extract of *R. communis* against *B. cucurbitae* and *B. zonata*; Table S2: TLC results of methanol fractions of *R. communis* using two different mobile phases; Table S3: TLC results of ethyl acetate fractions of *R. communis* using two different mobile phases; Table S4: TLC results of n-hexane fractions of *R. communis* using two different mobile phases.
