# Supplementary material for: Insecticidal Activity of Ricinus communis Leaf Extracts Against Bactrocera zonata and Bactrocera cucurbitae: Identification of Potential Bioactive Compounds
Source: Insects. 2026 Aug 5;17(8):811. doi: 10.3390/insects17080811 (PMC13513779; doi:10.3390/insects17080811)
Supplement: Supplementary file 1 [file insects-17-00811-s001.zip › insects-4439009-supplementary.pdf]

Supplementary Table S1. Treatment combinations, concentrations, and experimental design used for evaluation of the contact toxicity of crude ethanol extract of *R. communis* against *B. cucurbitae* and *B. zonata*.

Treatment Code	Extract preparation	Solvent used for dilution	Extract concentration (%)	Number of replications	Adults per replicate	Total adults tested per species
T1	Crude ethanol extract solution	Methanol	0.5	3	10	30
T2	Crude ethanol extract solution	Methanol	1.0	3	10	30
T3	Crude ethanol extract solution	Methanol	1.5	3	10	30
T4	Crude ethanol extract solution	Methanol	2.0	3	10	30
T5	Crude ethanol extract solution	Ethyl acetate	0.5	3	10	30
T6	Crude ethanol extract solution	Ethyl acetate	1.0	3	10	30
T7	Crude ethanol extract solution	Ethyl acetate	1.5	3	10	30
T8	Crude ethanol extract solution	Ethyl acetate	2.0	3	10	30
T9	Crude ethanol extract solution	*n*-Hexane	0.5	3	10	30
T10	Crude ethanol extract solution	*n*-Hexane	1.0	3	10	30
T11	Crude ethanol extract solution	*n*-Hexane	1.5	3	10	30
T12	Crude ethanol extract solution	*n*-Hexane	2.0	3	10	30
C1	Control	Methanol	0.0	3	10	30
C2	Control	Ethyl acetate	0.0	3	10	30
C3	Control	*n*-Hexane	0.0	3	10	30

Each treatment combination was tested on both *B. cucurbitae* and *B. zonata* separately. A total of 360 adults were tested per species (12 treatments × 3 replications × 10 adults). Solvent controls (C1–C3) were included for each dilution solvent.

Table S2. TLC results of methanol fractions of *R. communis* using two different mobile phases

Fractions	Mobile Phase	Spot	Rf value
Methanol Fraction (F1)	(Chloroform:n- hexane) 50:50 (V/V)	A	0.83
		B	0.39
		C	0.36
	(Ethyl acetate:methanol:chloroform)	A	0.22
		B	0.82
		C	0.22
Methanol Fraction (F2)	(Chloroform:n hexane) 50:50 (V/V)	A	0.11
		B	0.07
		C	0.10
	(Ethyl acetate:methanol:chloroform)	A	0.89
		B	0.91
		C	0.96
Methanol Fraction (F3)	(chloroform:n hexane) 50:50 (V/V)	A	0.09
		B	0.06
		C	0.06
	(Ethyl acetate:methanol:chloroform)	A	0.26
		B	0.23
		C	0.24

Table S3. TLC results of ethyl acetate fractions of *R. communis* using two different mobile phases

Fractions	Mobile Phase	Spot	Rf value
Ethyl acetate Fraction (F1)	(Chloroform:n hexane) 50:50 (V/V)	A	0.15
		B	0.21
		C	0.09
	(Ethyl acetate:methanol:chloroform)	A	0.07
		B	0.14
		C	0.07
Ethyl acetate Fraction (F2)	(Chloroform:n hexane) 50:50 (V/V)	A	0.5
		B	0.54
		C	0.56
	(Ethyl acetate:methanol:chloroform)	A	0.40
		B	0.31
		C	0.31
Ethyl acetate Fraction (F3)	(Chloroform:n hexane) 50:50 (V/V)	A	0.22
		B	0.10
		C	0.17
	(Ethyl acetate:methanol:chloroform)	A	0.25
		B	0.14
		C	0.74

Table S4. TLC results of *n*-hexane fractions of *R. communis* using two different mobile phases

Fractions	Mobile Phase	Spot	Rf value
n-hexane Fraction (F1)	(Chloroform:n hexane) 50:50 (V/V)	A	0.53
		B	0.52
		C	0.56
	(Ethyl acetate:methanol:chloroform)	A	0.93
		B	0.79
		C	0.73
n-hexane Fraction (F2)	(Chloroform:n hexane) 50:50 (V/V)	A	0.29
		B	0.07
		C	0.26
	(Ethyl acetate:methanol:chloroform)	A	0.44
		B	0.41
		C	0.41
n-hexane Fraction (F3)	(Chloroform:n hexane) 50:50 (V/V)	A	0.6
		B	0.49
		C	0.70
	(Ethyl acetate:methanol:chloroform)	A	0.42
		B	0.26
		C	0.35
